# A Review of Intracardiac Mass Cases Encountered Over a Two-Year Period

**DOI:** 10.7759/cureus.86619

**Published:** 2025-06-23

**Authors:** Shinji Akishima

**Affiliations:** 1 Cardiovascular Surgery, Ibaraki Prefectural Central Hospital, Kasama, JPN

**Keywords:** angiosarcoma, ball thrombus, combination therapy, intracardiac mass, myxoma, primary cardiac tumor, proton beam radiation, undifferentiated pleomorphic sarcoma

## Abstract

Intracardiac mass is a rare condition that poses significant diagnostic and therapeutic challenges. Regardless of the pathological diagnosis, these cases are often difficult to manage, as patients typically present with urgent clinical symptoms at their initial visit. Most symptoms result from blood flow disturbances caused by a large intracardiac tumor. Furthermore, patients face a high risk of thromboembolism.

Herein, we present five cases of intracardiac masses, including three malignant tumors, encountered over approximately two years. Each case had a different pathological diagnosis: angiosarcoma (n = 2), undifferentiated pleomorphic sarcoma (n = 1), benign myxoma (n = 1), and a simple ball thrombus (n = 1). Early and accurate diagnosis and treatment, ideally complete surgical resection, is the primary approach to managing intracardiac mass. However, extensive tumor resection can be difficult to achieve while preserving adequate cardiac function, especially in advanced-stage cases. In our series, surgical resection was not indicated for two patients (one with a ball thrombus and the other with angiosarcoma) due to impaired consciousness from multiple cerebral thromboembolism or advanced-stage sarcoma. The remaining three patients, diagnosed with myxoma, angiosarcoma, and undifferentiated pleomorphic sarcoma, underwent tumor excision. However, in the two malignant cases, early recurrence occurred, and postoperative management proved to be extremely challenging.

The three patients with primary cardiac sarcoma received radiation therapy combined with chemotherapy or multiple chemotherapy regimens as either primary or secondary treatment. Of the two patients diagnosed with angiosarcoma, one died two months after admission without undergoing surgery, while the other died 11 months following tumor resection, highlighting the limited effectiveness of available treatments. The remaining patient, diagnosed with undifferentiated pleomorphic sarcoma, also received combination therapy with radiation and chemotherapy. In this case, the primary in the left atrium and a solitary metastasis to the left adrenal gland were relatively controlled with proton beam therapy.

In cases of intracardiac mass, early diagnosis and prompt surgical excision are crucial. If the tumor is malignant, combined treatment with radiation and chemotherapy should be promptly considered as a second-line approach based on the tumor's characteristics and the extent of surgical resection. However, there are currently no curative treatments for either malignant or benign primary cardiac tumors other than complete tumor excision. Therefore, early-stage diagnosis is directly linked to improved prognosis.

## Introduction

Intracardiac masses, including benign tumors such as myxomas, malignant neoplasms such as sarcomas, and spherical thrombi, are rare, with an estimated incidence of approximately 0.02% in autopsy studies. These lesions pose significant diagnostic and therapeutic challenges due to their diverse characteristics and potential severity. Primary malignant cardiac tumors carry an especially poor prognosis, as recurrence and invasion are common, making them extremely difficult to manage. This report presents five cases of intracardiac masses, including three malignant tumors, encountered over approximately two years. Each case had different pathological diagnoses: angiosarcoma (n = 2), undifferentiated pleomorphic sarcoma (n = 1), benign myxoma (n = 1), and a simple ball thrombus (n = 1).

Both malignant and benign intracardiac masses can be challenging to manage, as patients often present with urgent clinical symptoms at diagnosis. Dyspnea, commonly resulting from blood flow obstruction caused by large intracardiac tumors, is frequently observed. Moreover, these patients are at high risk of thromboembolism. Therefore, early diagnosis and prompt treatment are essential. While complete resection is the preferred approach for cardiac tumors, extensive resection is challenging to perform while maintaining cardiac function, especially in advanced-stage cases. In addition, postoperative management of malignant tumors remains complex.

In this report, we describe cases of intracardiac masses encountered over a two-year period and discuss their diagnostic and therapeutic approaches. A literature review is also provided to support clinical insights and management strategies.

## Case presentation

Case 1

**Figure 1:**
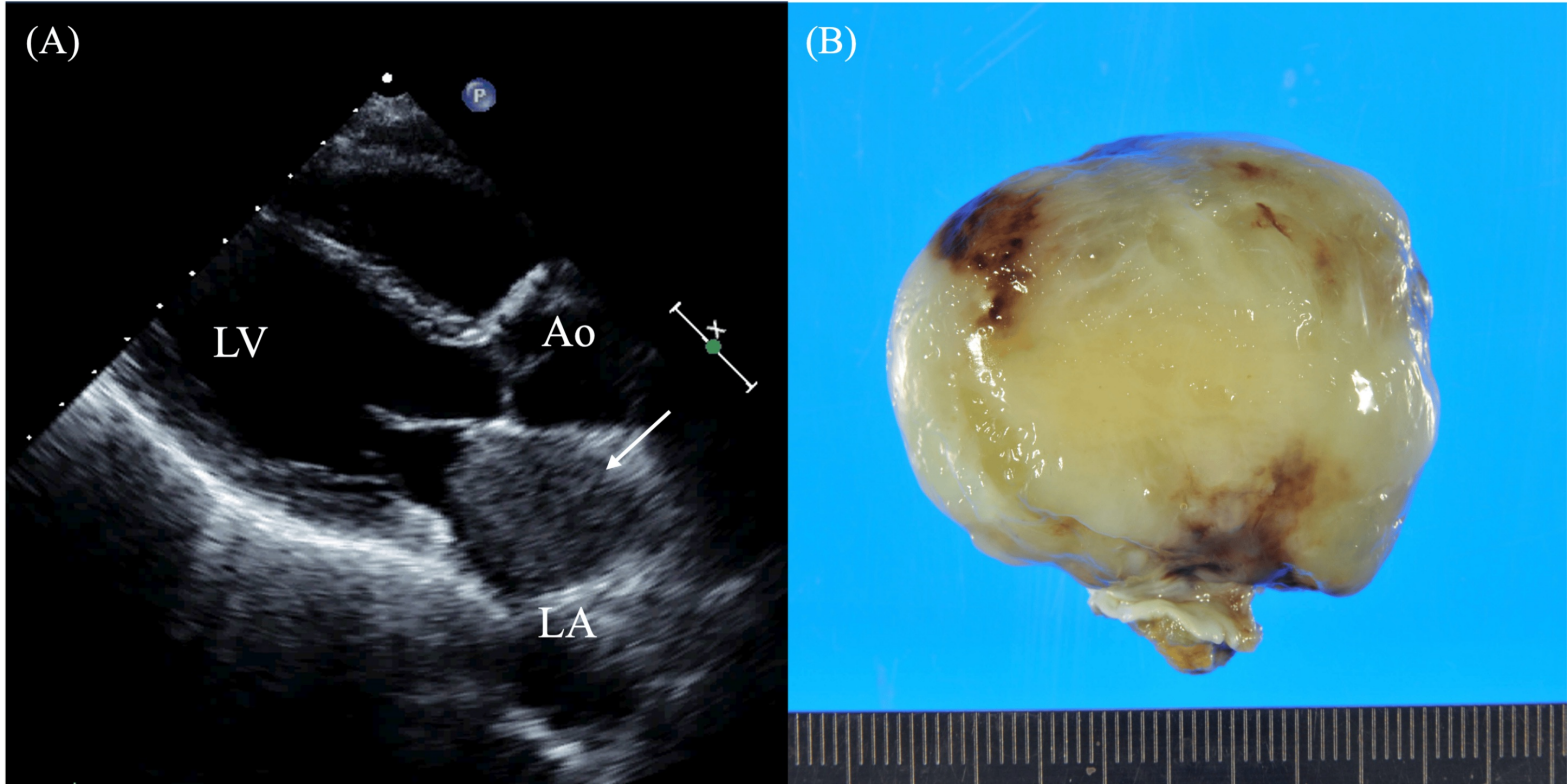
(A) UCG showing a large, mildly heterogeneous mass pedunculated from the atrial septum of the left atrium (arrow) on admission. (B) The excised tumor appeared soft and mucinous with a smooth surface (Case 1). UCG: ultrasound cardiography, LA: left atrium, LV: left ventricle, Ao: aorta.

A 58-year-old man had been experiencing mild dyspnea and palpitations during bathing for the past two months, initially suspected to be related to silicosis. Transthoracic ultrasound cardiography (UCG) revealed a slightly mobile mass in the left atrium (LA), pedunculated from the upper portion of the atrial septum (Figure 1A). The pedicle was short, preventing the tumor from reaching the mitral annulus. However, the LA was largely occupied by the large mass. On UCG, the tumor appeared as a soft, pedunculated mass with low echogenicity and a fluctuating, jelly-like surface, suggesting a gelatinous consistency. These features were consistent with a diagnosis of myxoma, a benign primary cardiac tumor. Further evaluation with computed tomography (CT) and magnetic resonance imaging (MRI) confirmed the presence of a myxoma with mildly heterogeneous internal content, consistent with the UCG findings. The patient’s general condition and circulatory status remained stable, and surgical extirpation of the tumor was performed as scheduled on the seventh day after admission. He was discharged on postoperative day 10 with a final pathological diagnosis of myxoma and continued follow-up in an outpatient setting (Figure 1B). No additional treatment was required.

Case 2

**Figure 2:**
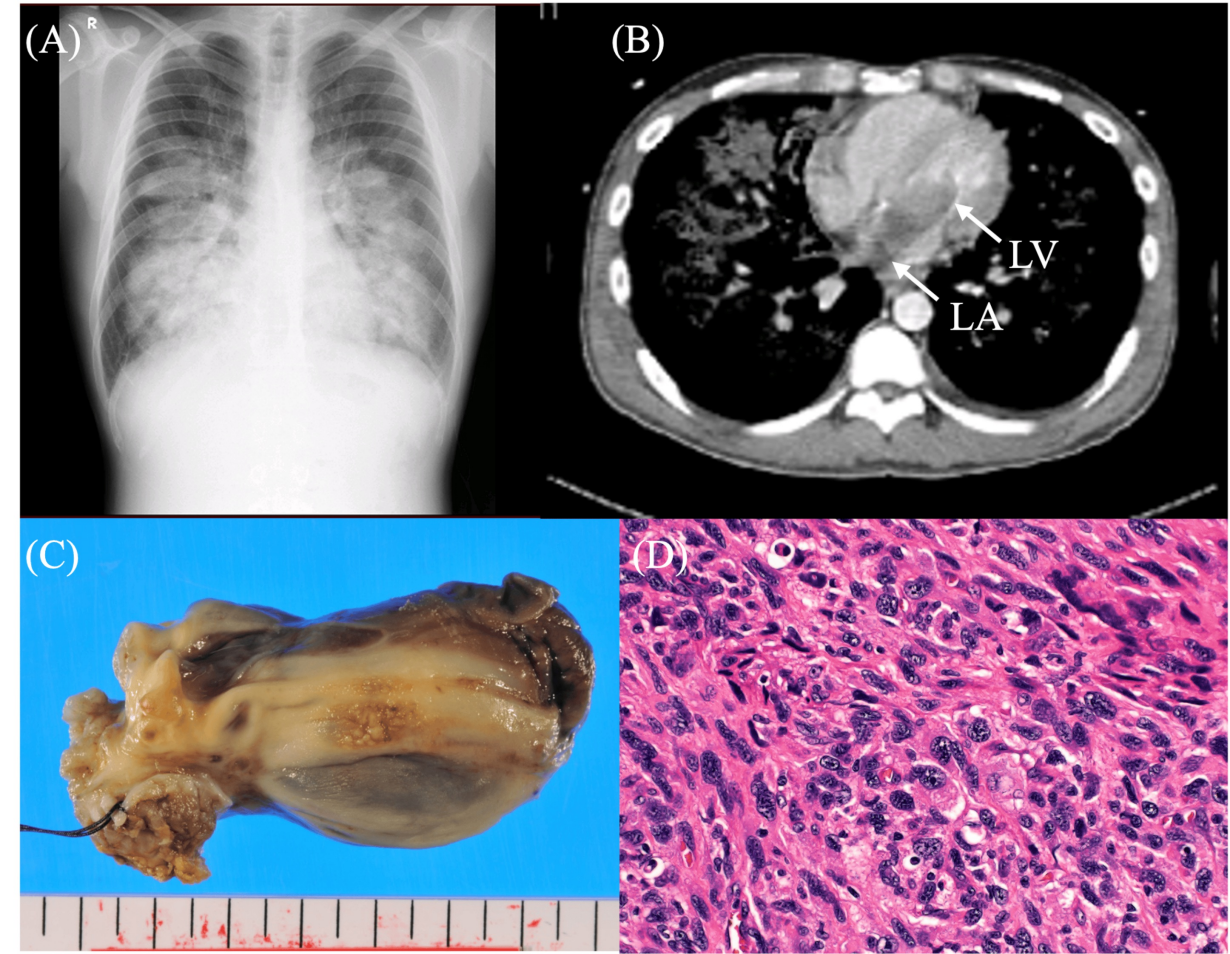
(A) Severe pulmonary congestion noted on chest roentgenography at admission. (B) Chest CT showing abnormal tumor shadow in the LA suspected to be incarcerated into the LV (arrow). (C) Excised cardiac tumor (75 x 37 x 30 mm) that occupied the LA cavity. (D) Pathological analysis confirmed an undifferentiated high-grade pleomorphic sarcoma, characterized by irregular spindle-shaped and multinucleated giant cells (hematoxylin and eosin staining) (Case 2). LA: left atrium, LV: left ventricle, CT: computed tomography.

A 41-year-old man presented with bilateral knee and ankle arthralgia that had persisted for approximately eight months. He was urgently transported to our institution due to acute and severe respiratory distress and hemoptysis that had begun about three hours prior. Upon arrival, the patient was in a state of shock. Chest roentgenography revealed severe pulmonary congestion (Figure 2A), and UCG showed a large mass in the LA that moved with the mitral valve leaflets and intermittently entered the left ventricle (LV). Color Doppler imaging demonstrated significant mitral valve flow obstruction, suggesting incarceration of the mass at the mitral annulus. Chest CT further revealed a tumor extending into the LA and LV (Figure 2B). Based on these findings, the patient was diagnosed with acute left heart failure caused by obstruction of cardiac blood flow due to the intracardiac mass.

**Figure 3:**
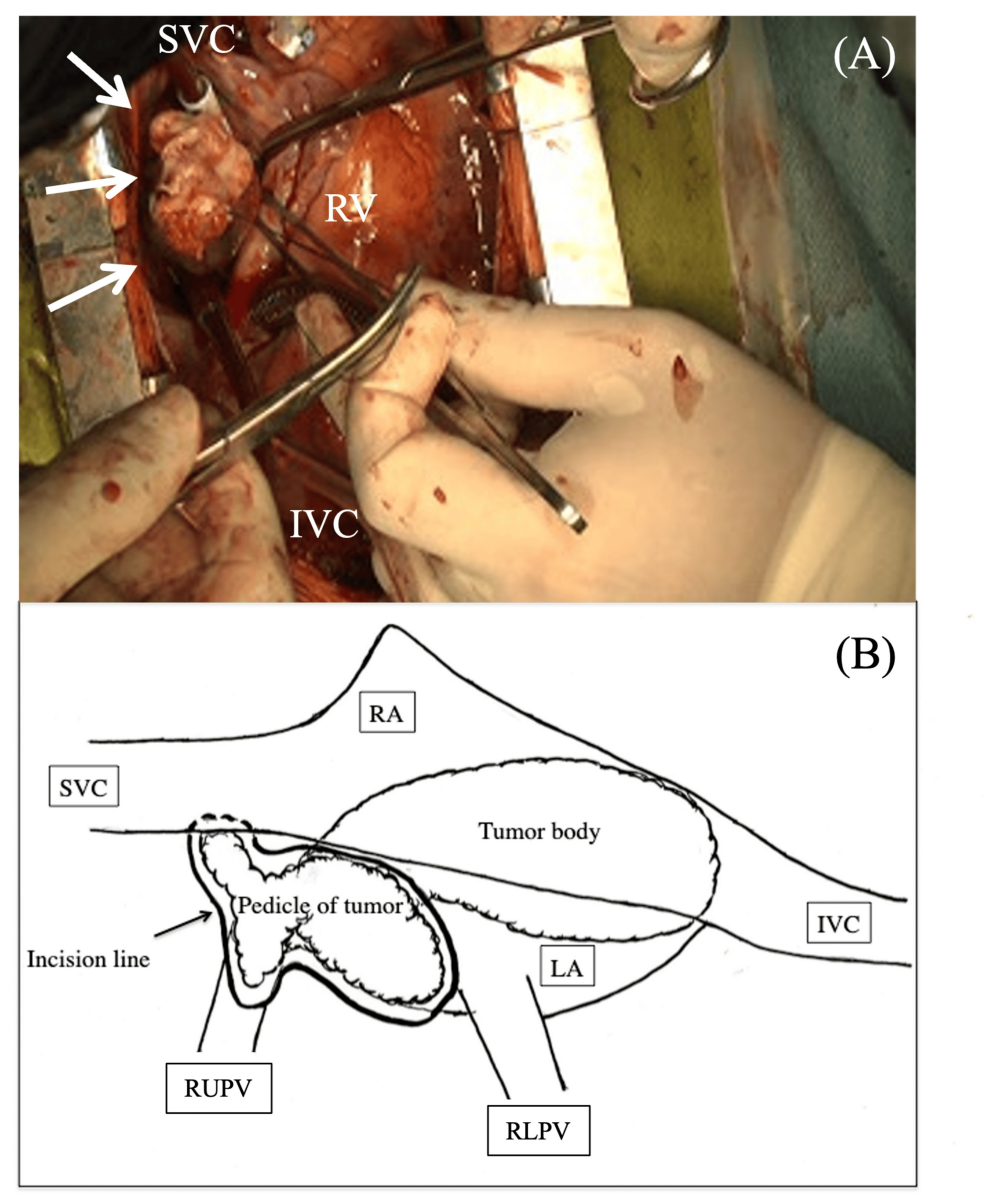
Intraoperative findings. (A) Intraoperative view showing the tumor being extirpated through a wide incision in the right-sided LA wall (arrow). (B) Intraoperative schema illustrating the tumor’s pedicle, which spread irregularly along the LA wall and extended into the right superior pulmonary vein. The main tumor body passed through the mitral annulus and reached the LV (Case 2). LA: left atrium, LV: left ventricle, SVC: superior vena cava, IVC: inferior vena cava, RV: right ventricle, RA: right atrium, RUPV: right upper pulmonary vein,  RLPV: right lower pulmonary vein.

An emergency surgery under extracorporeal circulation was performed. Owing to the tumor's location and pedicle attachment, the right LA was approached via a wall incision in the right upper pulmonary vein. The tumor pedicle originated from the right upper and posterior LA wall and extended into the right upper pulmonary vein (Figure 3). Intraoperatively, the tumor had an irregular shape, heterogeneous appearance, and varying consistency, with an extensive stalk-like spread, raising strong suspicion of malignancy. We attempted maximal resection to the best of our ability. Macroscopically, we believe that nearly the entire tumor was removed. However, at the periphery of the right upper pulmonary vein, limited access from the pericardium and mediastinum prevented achieving sufficient margins. Intraoperative rapid pathological diagnosis was not performed, as the procedure took place during nighttime hours. The mitral valve was intact morphologically and functionally. The large defect in the LA wall was reconstructed using a thin Gore-Tex sheet.

The final pathological diagnosis was undifferentiated pleomorphic sarcoma (formerly referred to as malignant fibrous histiocytoma), with invasion into the atrial muscular layer (Figures 2C, 2D). The first local recurrence was detected in the posterior wall of the LA seven months after the surgery. Thus, proton beam radiotherapy was selected, and a dose of 75 Gy was administered in 30 fractions for 45 days. Two months after the radiotherapy, no residual tumor was visible on CT imaging. However, six months later, a second local recurrence at a different site in the LA and a distant metastasis to the left adrenal gland were identified. Proton beam radiation was again employed to manage both lesions while preserving the left kidney function: 60 Gy in 30 fractions was delivered to the LA and 46 Gy in 23 fractions to the adrenal gland. Moreover, chemotherapy with pazopanib hydrochloride (800 mg/day), a tyrosine kinase inhibitor (molecular-targeted drug), was administered in combination with radiotherapy.

At eight months following the second radiotherapy (27 months post-surgery), no active tumors were detected on any imaging tests. However, after a further two months without effective treatment, a third local recurrence was identified in the LA. The patient was unable to return home due to the recurrence, which was attributed to reaching the permissible maximum radiation dose and the limited effectiveness of chemotherapy.

Case 3

**Figure 4:**
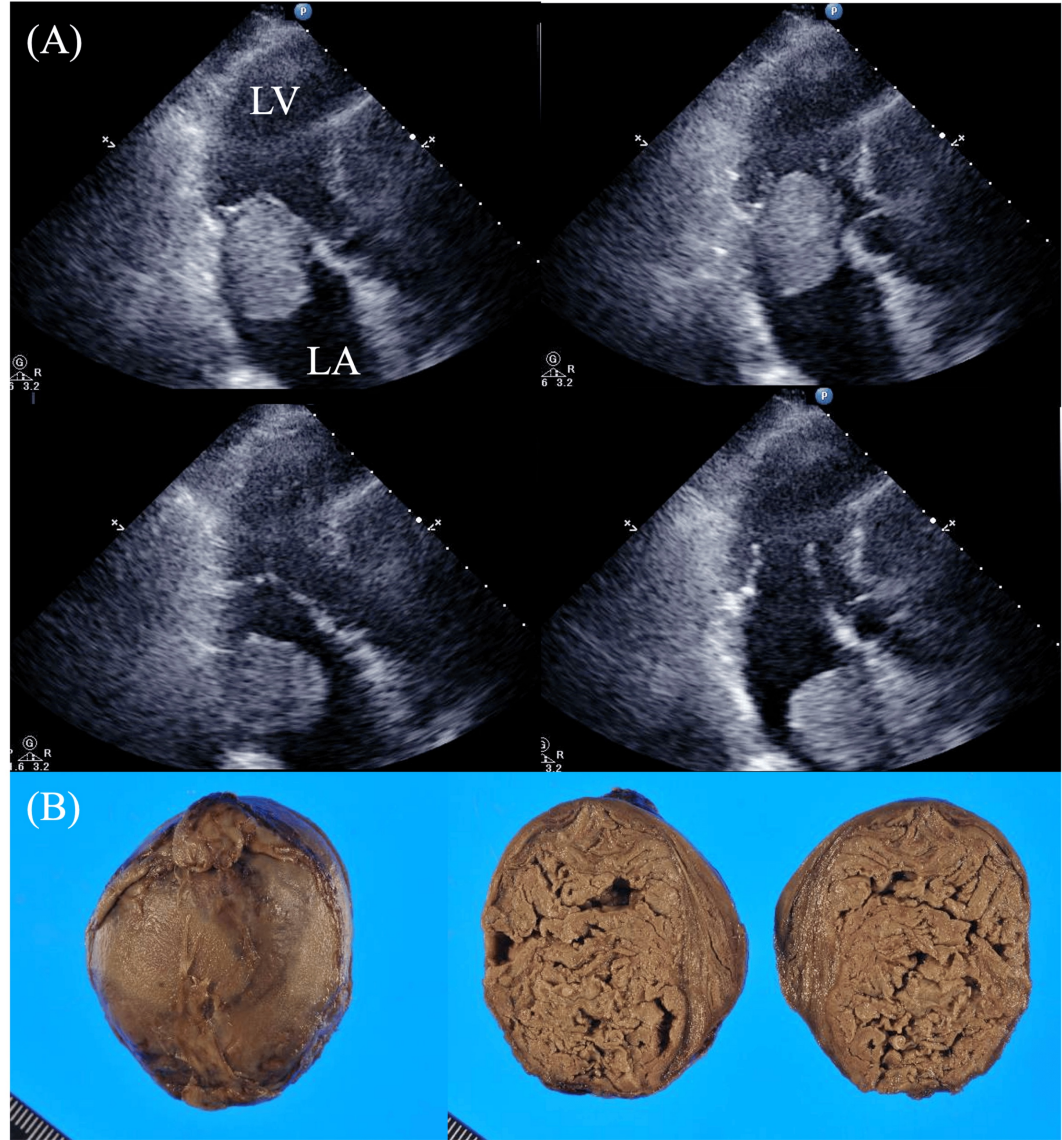
(A) UCG on admission revealed a spherical, freely mobile mass within the LA. (B) Autopsy identified a smooth-surfaced ball thrombus in the LA, about 40 mm in diameter (Case 3). UCG: ultrasound cardiography, LA: left atrium, LV: left ventricle.

**Figure 5:**
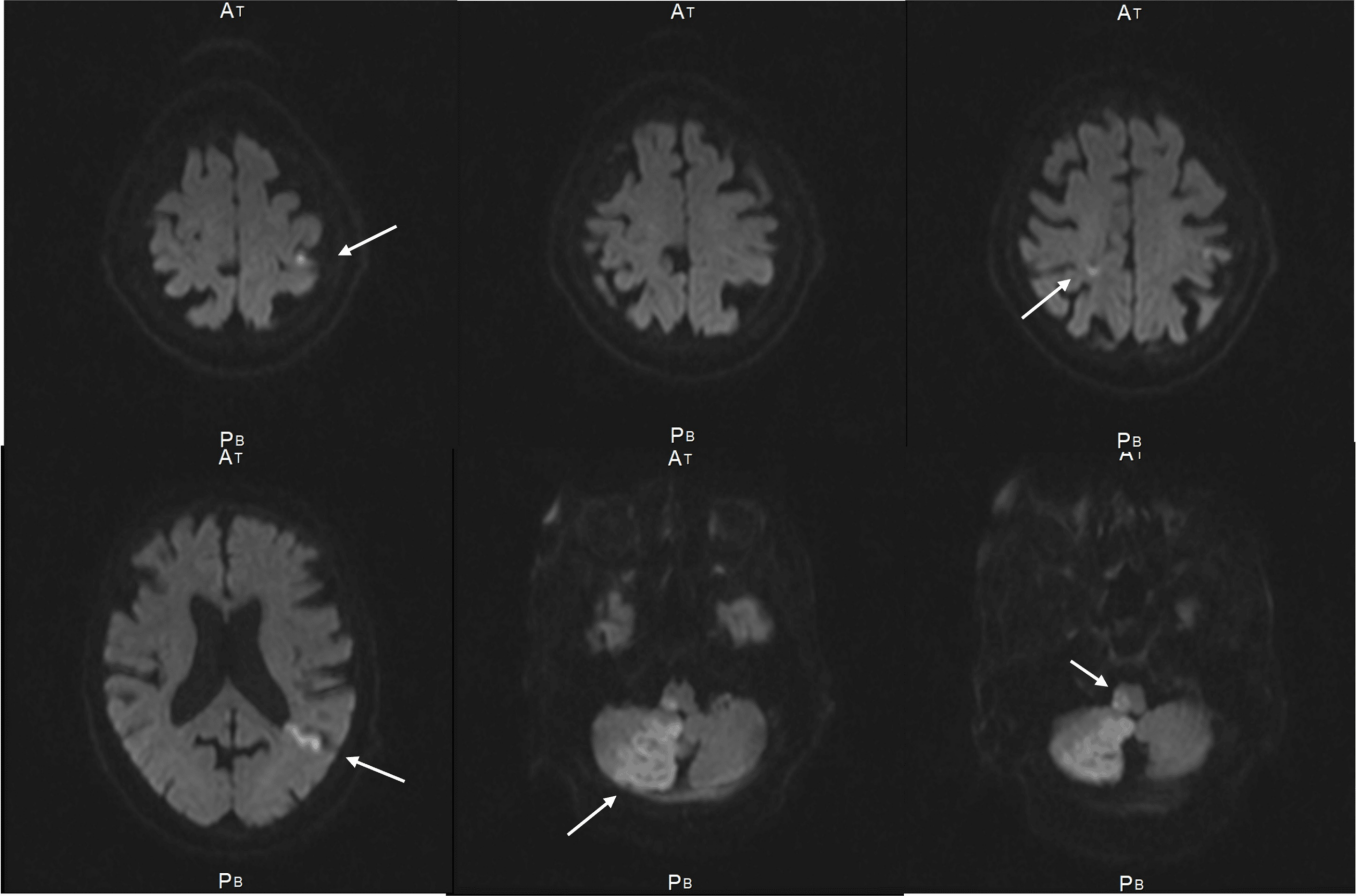
Multiple fresh brain infarctions (Case 3). MRI with diffusion-weighted imaging at initial consultation showed multiple acute infarctions in the right cerebellum, right brainstem, and bilateral temporal/parietal lobes (arrows), contributing to the patient's impaired consciousness. MRI: magnetic resonance imaging.

The third case involved an 81-year-old woman, identified two months after Case 2, who was found to have a floating mass in her LA. She had been diagnosed with atrial fibrillation three years prior and had been on medication since then. Three days before admission, she suddenly experienced dizziness, nausea, and palpitations, and was referred to our hospital from a nearby clinic. UCG revealed a spherical, uniformly echogenic mass in the LA, approximately 35 mm in diameter. The mass was freely mobile within the LA, but due to its size and mobility, there was a risk that it could become incarcerated in the LV (Figure 4A). Despite the atrial fibrillation, the patient's circulatory status remained stable. Emergency surgery was considered; however, moderate disturbance of consciousness had already developed by the time of evaluation. MRI revealed multiple acute cerebral infarctions in the right cerebellum, right brainstem, and bilateral temporal and parietal lobes (Figure 5). No interventional treatment was administered. The patient’s neurological condition deteriorated progressively, and she died on the fifth day of admission. An autopsy confirmed that the mass in the LA was a ball thrombus, characterized by its spherical shape and smooth surface (Figure 4B).

Case 4

**Figure 6:**
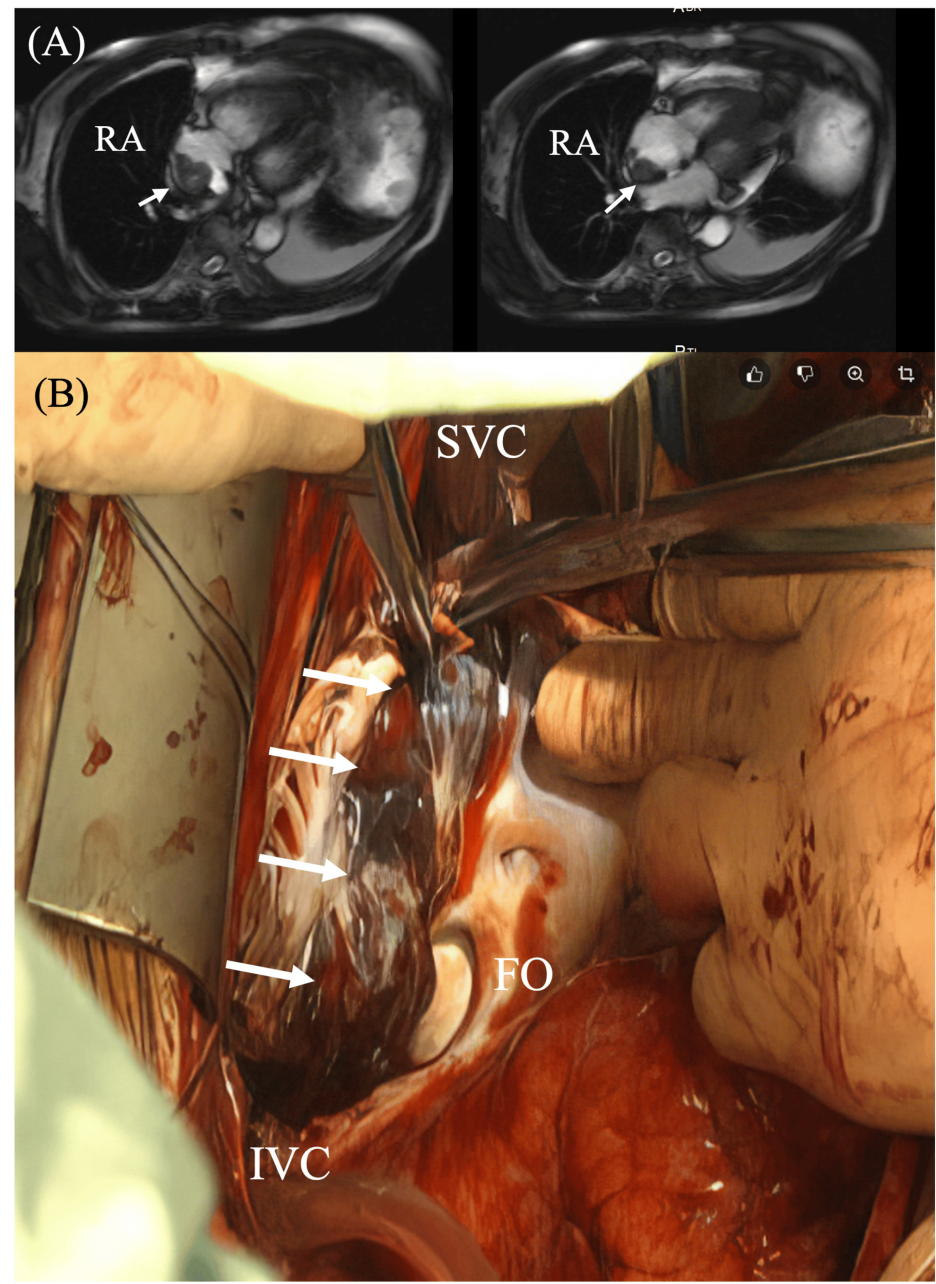
(A) Preoperative MRI showing a mass extensively adherent to the posterior and inferior walls of the RA (arrow). (B) Intraoperative view showing a tumor invading all layers of the atrial wall (arrow) (Case 4). RA: right atrium, MRI: magnetic resonance imaging, SVC: superior vena cava, IVC: inferior vena cava, FO: fossa ovalis.

The patient was a 74-year-old man who presented with dyspnea unrelated to exertion, which had persisted for approximately one month. Imaging studies revealed cardiomegaly with moderate pericardial effusion. Although myocardial thickening was observed, LV motion remained largely normal. After admission, bloody pericardial effusion was identified, and laboratory tests showed an elevated cancer antigen 19-9 level. MRI revealed a broad-based, extensive abnormal shadow in the right atrium (RA) (Figure 6A). The lesion showed heterogeneous signal intensity on both T1- and T2-weighted imaging, suggestive of tumor tissue, necrosis, and hemorrhage, findings highly indicative of angiosarcoma. Furthermore, positron emission tomography (PET) revealed abnormal uptake limited to the heart, supporting a diagnosis of a primary cardiac tumor.

A scheduled surgical resection was performed. Intraoperatively, the tumor was found to be extensively adherent to the posterior RA wall and extended into the inferior vena cava (Figure 6B). The tumor, along with the involved portion of the RA wall and parietal pericardium, was resected with adequate margins. The resulting defect was reconstructed using a thin Gore-Tex sheet. Despite the pathological diagnosis of angiosarcoma, the patient’s postoperative course was relatively stable and smooth. However, 1.5 months after surgery, he developed recurrent pericardial and pleural effusion. Pathological analysis reconfirmed malignancy. Weekly chemotherapy with paclitaxel was initiated and was effective in controlling the effusions for about two months. Unfortunately, treatment was discontinued due to the progression of pleural effusion and the development of peripheral neuropathy. Subsequent imaging showed marked pericardial dissemination, prompting a switch to pazopanib as next-line therapy. Despite this, the tumor progressed rapidly. The patient developed massive pleural effusion, and pleural adhesion procedures were ineffective. He died 11 months after tumor resection. An autopsy revealed not only massive pericardial and pleural effusion but also peritoneal dissemination and metastasis to the left adrenal gland.

Case 5

**Figure 7:**
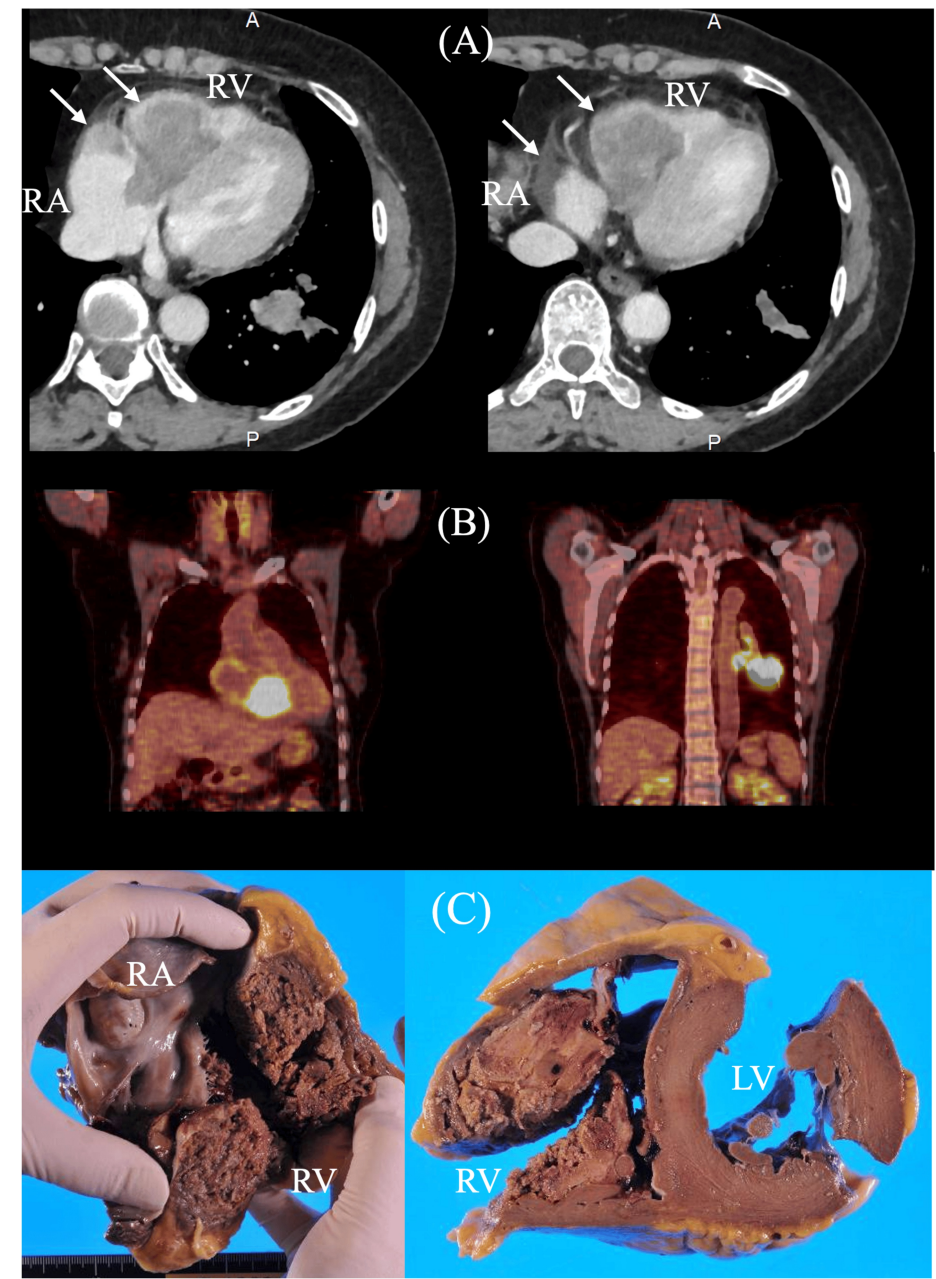
(A) Contrast-enhanced CT at initial presentation showing the right ventricle (RV), including the tricuspid annulus, completely occupied by a tumor mass (arrow). (B) PET scan revealing abnormal high uptake in the left lung, indicating distant metastasis. (C) Autopsy demonstrating massive tumor involvement throughout the right-sided cardiac chambers (Case 5). RV: right ventricle, RA: right atrium, CT: computed tomography, PET: positron emission tomography.

The patient was a 63-year-old woman with primary cardiac angiosarcoma. She initially presented with a persistent cough and low-grade fever that had lasted for approximately three months. Two weeks prior to her hospital visit, she experienced a sudden worsening of dyspnea. Imaging studies revealed an abnormal shadow in the left lung and the right side of the heart. The shadow occupied much of the right ventricle (RV), originating just above the tricuspid annulus in the RA (Figures 7A, 7B). At that point, surgical intervention was not considered a viable life-saving option. Complete tumor resection would have severely compromised cardiac function, and the presence of widespread metastases rendered the procedure inappropriate. Laboratory findings showed elevated levels of neuron-specific enolase, a tumor marker. A pathological diagnosis of angiosarcoma was confirmed via catheter biopsy. Subsequently, chemotherapy with pazopanib hydrochloride at a dose of 800 mg/day was administered 27 days after admission, during which thrombocytopenia developed. Thus, the patient was simultaneously managed with radiation therapy at a dose of 45 Gy/15 Fr. However, none of the treatments were considerably effective, and the patient died two months after admission due to pan-peritonitis secondary to pancreatic metastasis (Figure 7C).

## Discussion

Intracardiac masses are a rare condition that requires various treatment approaches [1]. In particular, the incidence of primary cardiac tumors is approximately 0.02%, with about 80% being benign and 20% malignant. The distribution by location is approximately 60% in the LA, 20% in the RA, 15% in the ventricles, and 5% in the valves [1,2]. Their pathological characteristics (benign vs. malignant) pose treatment challenges, especially in malignant cases, where the prognosis is extremely poor [1-3]. Moreover, even cardiogenic masses, such as a ball thrombus, can notably affect cardiac function. Therefore, patients often present with unstable circulatory dynamics at the initial visit, and treatment, including surgical excision, should be initiated promptly. In addition, obtaining a definitive diagnosis is extremely important, primarily through imaging studies. However, we acknowledge that not all cases underwent comprehensive imaging examinations, such as cardiac MRI and PET. For example, in Case 2, a definitive pathological diagnosis could not be confirmed using CT alone. This limitation highlights the importance of integrating clinical findings and other diagnostic modalities when determining initial treatment based on the pathology of the mass.

However, the reason for striving to obtain a pathological diagnosis preoperatively in patients with intracardiac masses, even in urgent clinical situations, lies in the concept of tumor doubling time (TDT) [4,5]. TDT refers to the time it takes for a tumor to double in size, reflecting its growth rate and clinical behavior. Benign tumors generally have longer TDTs, grow slowly, and present with minimal symptoms, making them easier to excise with lower recurrence rates. In contrast, malignant tumors typically have shorter TDTs, grow rapidly, and exhibit aggressive behavior, leading to early symptoms and a higher risk of metastasis. This necessitates more extensive surgical margins and complex treatment. Malignant primary cardiac sarcomas, such as angiosarcoma and undifferentiated pleomorphic sarcoma, often have extremely short TDTs, ranging from weeks to months. Accurate preoperative diagnosis, aided by imaging studies like MRI and PET scans, is crucial in guiding surgical and postoperative management. Understanding TDT helps clinicians predict tumor progression and optimize treatment strategies.

The current study reported five cases of intracardiac masses encountered over approximately two years and treated based on their pathological diagnoses (Table 1). As noted previously, most patients were in a state of circulatory urgency at the time of their initial diagnosis. Thus, a prompt diagnosis was made, and an appropriate treatment plan was implemented. However, treatment strategies were extremely complex, particularly given our limited experience in managing such tumors.

**Table 1:** Summary of the five reported cases of intracardiac masses. LA: left atrium, RA: right atrium, LV: left ventricle.

Case no.	Age	Gender	Diagnosis	Pathologic diagnosis	Location	Initial symptoms	Duration until the first visit	Surgical resection	Radiation therapy	Chemotherapy	Outcome	Duration since the first visit
1	58	Male	Myxoma	Benign	LA	Palpitation	2 months	Performed (urgent )	-	-	Alive	>5 years
2	41	Male	Undifferentiated pleomorphic sarcoma	Malignant	LA	Dyspnea, hemoptysis, arthralgia	1 day 3 hours (8 months)	Performed (emergent)	Performed (proton)	Performed (pazopanib)	Dead	31 months
3	81	Female	Ball thrombus	Benign	LA	Dizziness, nausea	3 days	Inoperable	ー	ー	Dead	5 days
4	74	Male	Angiosarcoma	Malignant	RA	Dyspnea	1 month	Performed (urgent)	Not indicated	Performed (paclitaxel, pazopanib)	Dead	11 months
5	63	Female	Angiosarcoma	Malignant	RV	Cough, dyspnea	3 months 2 weeks	Inoperable	Performed	Performed (pazopanib)	Dead	2 months

In all cases, transthoracic or transesophageal echocardiography provided essential information that helped us identify the nature of the tumor (a neoplastic mass) and make an approximate diagnosis (benign vs. malignant). In addition, CT scans and other imaging methods provided details on the circulatory disturbance caused by the masses and their metastasis throughout the body. Based on these findings, we were able to estimate how much time remained before surgical excision was necessary. However, MRI and PET were not always performed due to time constraints and facility limitations. In any case, regardless of the pathological diagnosis, the initial treatment requires the complete excision of the intracardiac mass. Nevertheless, in cases of primary cardiac tumors, there are limitations to surgical resection due to advanced local spread and further invasion into critical intracardiac structures. The basic approach is to operate with as much margin as possible and aim for complete resection, even if only visually confirmed. Therefore, the surgeon should remove as much of the tumor as possible while preserving adequate cardiac function to allow successful weaning from extracorporeal circulation.

The second, fourth, and fifth cases in our series involved sarcomas as primary malignant cardiac tumors. In the second case, the tumor was grossly resected under visual guidance; however, the right pulmonary vein was extensively involved, and the postoperative pathology revealed a positive margin at the resection line. It was assumed that curative resection would have required more extensive surgery, including removal of part of the right lung. In the fourth case, the tumor filled the RA, extending from the atrial roof to the superior vena cava, and bloody pericardial effusion was noted. Thus, tumor resection was clearly noncurative. In the fifth case, the tumor was extremely large, filling the right ventricle and engulfing the tricuspid valve. Surgical resection was not performed, as it was deemed to be only diagnostic rather than therapeutic.

In the first case, which involved a benign LA myxoma, the tumor required early and extensive resection similar to that for a malignant cardiac tumor due to its size, mobility, and the associated risk of mitral valve function impairment and secondary embolism caused by tumor tissue or thrombus induction. In addition, annual postoperative follow-up was also required, as recurrence can occur if the stump is not completely resected.

The third case, which involved a spherical thrombus in the LA, was diagnosed in a relatively rare manner, initially resembling a suspected cardiac tumor. A primary thrombus had developed in a thrombophilic intracardiac environment, resulting in a tumor-like ball thrombus. However, similar to the myxoma in the first case, this condition should be considered clinically malignant due to its large size and high mobility, which posed a significant risk for severe left heart dysfunction from its insertion into the mitral valve, as well as major embolization from the primary and secondary thrombi. In fact, the patient had already developed impaired consciousness due to multiple cerebral infarctions, likely caused by thromboembolism. Furthermore, surgical removal could not be performed due to the rapid progression of the disease, ultimately resulting in the patient's death.

Here, the treatment strategies for malignant primary cardiac tumors are considered. As mentioned earlier, the pathological diagnosis of malignant primary cardiac tumors is almost always sarcoma. Sarcomas remain challenging to manage regardless of their location, and complete surgical removal is the main treatment option [1,3,6-8]. However, compared to other organs, it is particularly difficult to completely resect cardiac tumors with sufficient margins. This difficulty can be attributed to several reasons. First, resection significantly affects cardiac function due to the involvement of vital intracardiac structures. Second, it is challenging to perform extensive resection followed by reliable reconstruction. Third, it is necessary to promptly restore and maintain adequate cardiac function immediately after resection. As with all malignant tumors, the most critical factor is how early the tumor can be detected and diagnosed. Notably, accurate and complete resection of the tumor is essential for improving prognosis and achieving a potential cure [9-11].

Secondary treatments following tumor resection are discussed next, with a focus on TDT as a critical factor. The five reported cases of intracardiac masses demonstrated diverse pathological diagnoses, including three cases of primary cardiac sarcoma, highlighting the significant challenges involved in their treatment and management. At that time, there were only a few cases, either from our own experience or from other reports, with both definitive treatment methods and favorable prognosis. Since our experience with this case series more than five years ago, we have encountered only three cases of intracardiac masses, all of which were benign tumors such as myxomas. It was quite unusual to have managed so many cases of intracardiac tumors over a span of about two years, and unfortunately, I have not been able to leverage that experience further. As previously discussed, effective treatment strategies for sarcomas, the main type of malignant primary cardiac tumor, remain limited. Sarcomas still carry an ultimately poor prognosis due to their extremely short TDT [12,13].

Paclitaxel, commonly used for angiosarcoma, and pazopanib, often administered for undifferentiated pleomorphic sarcoma, are now considered first-line drug treatments for sarcomas. These tumors often have poor vascular structure and limited statistical insights derived from genetic analysis [1,14]. However, there are still few reports describing highly effective chemotherapeutic regimens [1,2,6,8]. Nonetheless, there is growing optimism regarding the potential of multi-tyrosine kinase inhibitors and MDM2 inhibitor-based molecular-targeted drugs, even today. We anticipate that a substantial body of encouraging results and supporting evidence will become clearly demonstrated in the future [1,6,8].

By contrast, some studies have reported that radiation therapy has specific therapeutic effects [15,16]. In particular, irradiation of primary tumors or solitary distant metastatic sites using heavy particle beams, such as proton beams, can temporarily eliminate tumors for a defined period [3]. However, radiation therapy has significant limitations due to the maximum allowable cumulative dose. Even if temporary complete remission is achieved, most tumors tend to recur in adjacent areas, and the organ is considered in remission, not completely cured. Additionally, in several cases, functional deterioration of the targeted area can occur due to radiation-induced ablation. In cardiac cases, this may lead to functional impairment. Even more than five years after encountering our series of cases, the principle remains unchanged: only complete surgical resection has been associated with improved prognosis, and no definitive or effective secondary treatment has been established since then [9-11].

In Case 2, the combination of chemotherapy with pazopanib and proton beam irradiation prolonged the patient’s life by more than 2.5 years after excision surgery. In Case 4, paclitaxel was combined with pazopanib. In Case 5, pazopanib was combined with linear accelerator radiation. However, none of these treatments appeared effective in suppressing even pericardial effusions or pleural effusions. Therefore, even combination therapy is less effective in cases of advanced-stage cardiac sarcoma.

Most cardiovascular surgeons are not specialized in the treatment of tumors, making collaboration with an oncology team extremely important in managing malignant primary cardiac tumors. Again, it is not an exaggeration to state that the prognosis is significantly influenced by how early the tumor is detected and diagnosed. With the accumulation of case data on these extremely rare cardiac tumors, and the recent improvements in diagnostic imaging, particularly UCG, which can be performed immediately, we are now in a better position to aim for appropriate, extensive excision and complete resection of the tumor from the point of initial diagnosis. Moving forward, the focus must shift toward recognizing cardiac tumors before they result in circulatory compromise.

In three of the reported malignant cases, respiratory symptoms such as coughing began approximately 1-3 months prior to diagnosis. These symptoms were caused by the tumor's impact on circulation. However, if a detailed examination had been performed at that stage, it might have been possible to reach a diagnosis of a cardiac tumor earlier. In Case 2, which involved malignant histiocytoma, the patient had experienced unexplained pain or discomfort in the knees and other joints well before the diagnosis. Interestingly, these symptoms resolved after the tumor was surgically removed or when its volume was reduced by radiotherapy. The patient was also aware that the joint discomfort returned before the disease recurrence. Although this observation is retrospective, laboratory tests showed elevated levels of certain tumor markers and interleukins, particularly interleukin-6. This finding suggests that specific tumor markers and interleukins may be associated with tumor activity and could potentially induce paraneoplastic syndromes.

## Conclusions

Within approximately two years, five cases of intracardiac tumors were encountered, each with a different pathological diagnosis. In all cases, the patients exhibited clinical symptoms resulting from disturbances in circulatory dynamics. Furthermore, the tumors were highly complex, requiring acute management of the presenting condition and long-term treatment planning for the primary tumor. Even today, only a few reports have demonstrated the effectiveness of radiation or chemotherapy for cardiac tumors, particularly sarcomas. Therefore, making a diagnosis at the earliest possible stage, followed by initial complete resection, remains the recommended strategy for achieving a favorable prognosis.
